# Selection of Reference Genes for RT-qPCR Analysis in the Monarch Butterfly, *Danaus plexippus* (L.), a Migrating Bio-Indicator

**DOI:** 10.1371/journal.pone.0129482

**Published:** 2015-06-01

**Authors:** Huipeng Pan, Xiaowei Yang, Keith Bidne, Richard L. Hellmich, Blair D. Siegfried, Xuguo Zhou

**Affiliations:** 1 Department of Entomology, University of Kentucky, Lexington, Kentucky, United States of America; 2 Department of Entomology, Cornell University, Ithaca, New York, United States of America; 3 Corn Insects and Crop Genetics Research Unit and Department of Entomology, Iowa State University, Ames, Iowa, United States of America; 4 Department of Entomology, University of Nebraska, Lincoln, Nebraska, United States of America; Institute of Vegetables and Flowers, Chinese Academy of Agricultural Science, CHINA

## Abstract

Reverse transcriptase-quantitative polymerase chain reaction (RT-qPCR) is a powerful technique to quantify gene expression. To facilitate gene expression study and obtain accurate results, normalization relative to stably expressed reference genes is crucial. The monarch butterfly, *Danaus plexippus* (L.), is one of the most recognized insect species for its spectacular annual migration across North America. Besides its great voyages, *D*. *plexippus* has drawn attention to its role as a bio-indicator, ranging from genetically modified organisms (GMOs) to natural ecosystems. In this study, nine reference genes from *D*. *plexippus* genome were selected as the candidate reference genes. The expression profiles of these candidates under various biotic and abiotic conditions were evaluated using the four readily available computational programs, *BestKeeper*, *Normfinder*, *geNorm*, and *ΔC_t_* method, respectively. Moreover, *RefFinder*, a web-based computational platform integrating the four above mentioned algorisms, provided a comprehensive ranking of the stability of these reference genes. As a result, a suite of reference genes were recommended for each experimental condition. Specifically, *elongation factor 1α* (*EF1A*) and *ribosomal protein 49* (*RP49*) were the most stable reference genes, respectively, under biotic (development, tissue, and sex) and abiotic (photoperiod, temperature, and dietary RNAi) conditions. With the recent release of a 273-million base pair draft genome, results from this study allow us to establish a standardized RT-qPCR analysis and lay a foundation for the subsequent genomic and functional genomic research in *D*. *plexippus*, a major bio-indicator and an emerging model for migratory animals.

## Introduction

The advent of next-generation sequencing technologies has led to a significant increase in transcriptomic and genomic output for various organisms [1–3]. Validation of gene expression has become a standard for reporting and assessing the quality of these transcriptomic and genomic resources [4, 5]. Reverse transcriptase-quantitative polymerase chain reaction (RT-qPCR) is a powerful technique to target and quantify gene expression [6], however, there remain limitations can significantly influence the normalization of gene expression, such as variations in RNA extraction, RNA quality and integrity, cDNA quality, and PCR efficiency [7–10]. In RT-qPCR, a commonly used technique to normalize the gene expression data is to introduce one or multiple reference genes, which are stably expressed across various experimental conditions and serve as the internal control [6, 9, 10]. Although these reference genes have been defined functionally as ‘constitutively expressed to maintain cellular function’, they do not necessarily meet prerequisites for a good reference gene that can be ‘expressed at constant levels across various biotic and abiotic conditions [4, 8, 10]. Many studies have proved that some commonly used reference genes express differentially across different experimental conditions; researchers have documented that multiple reference genes should be used for accurate normalization [4, 8, 9, 11, 12].

RNA interference (RNAi) is a biological process in which RNA molecules inhibit gene expression, typically by causing the destruction of specific mRNA molecules. In view of its high sequence specific reality, RNAi has drawn great attention from the crop protectors who recognized it as a novel and environmentally friendly ways for insect pest control [13, 14]. Recently, RNAi-based transgenic crops targeting insects have been developed [15–17]. One of the major ecological concerns with respect to the bio-safety of transgenic crops on the environment is their potential effects on non-target organisms [14]. The monarch butterfly is recognized as an important symbol of biodiversity and had been selected as a surrogate species for the risk assessment of *Bacillus thuringiensis* (*Bt*) and RNAi-based transgenic crops. Given the nature of RNAi mechanisms, non-target effects could occur via the modulation of gene expressions in these organisms [13]. Therefore, RT-qPCR will be a major research tool to evaluate potential non-target effects of this new biotechnology.

The monarch butterfly, *Danaus plexippus *(L.) (Lepidoptera: Danainae), is famous for its late summer/autumn southward migration from the United States and southern Canada to Mexico, and northward return in spring, which occurs over the lifespan of three to four generations of the butterfly [18]. In addition to this migration, *D*. *plexippus* has drawn attention as a surrogate species for the ecological risk assessment of genetically modified organisms (GMOs) [19–22] and as an eco-indicator for global climate change [23, 24]. The recent release of a 273-million base pair draft genome, including 16,866 protein-coding genes [25], provides an unprecedented opportunity to investigate the genetic basis governing monarch migration, including but not limited to genes involved in their migratory behavior, circadian rhythm, juvenile hormone modulation, and warning coloration [25–27]. Recent studies have identified the specific areas in the genome of the monarch that regulate migration. No genetic difference was observed between migrating and non-migrating monarchs but certain genes are expressed solely in migrating monarchs [25, 27].

The objective of this study was to determine suitable reference genes with stable expression in *D*. *plexippus* across various biotic and abiotic conditions. Here, nine reference genes from *D*. *plexippus* genome were selected as candidate reference genes [25], including *elongation factor 1α* (*EF1A*), *glyceralde hyde-3-phosphate dehydro-genase* (*GAPDH*), *nicotinamide adenine dinucleotide* (*NADH*), *cyclophilins A* (*CypA*), *ribosomal protein S5* (*RPS5*), *ribosomal protein 49* (*RP49*), *vacuolar-type H* ^*+*^ *-ATPase* (*v-ATPase*), *28S ribosomal RNA* (*28S*), and *18S ribosomal RNA* (*18S*). All of these candidates have been used previously as endogenous references for gene expression analyses [4, 8–12]. Stability of these genes was investigated under three biotic (developmental stage, tissue type, and sex) and three abiotic (temperature, photoperiod, and dietary RNAi) conditions. As a result, different sets of reference genes were recommended for each experimental condition. To validate the recommendations, effectiveness of these candidates were further examined by RT-qPCR analysis of a circadian clock gene *timeless* (*tim*) [28].

## Materials and Methods

### Ethics Statement

Eggs of *Danaus plexippus* were collected from common milkweed, *Asclepias syriaca*, near Ames, Iowa spring and summer 2013. No specific permit was required for the field collection. *Danaus plexippus* is a common American butterfly species in the United States. The permit to move live plant pests, noxious weeds, and soil were authorized by the United States Department of Agriculture Animal and Plant Health Inspection Service (Permit number: P526P-13-03521). Four to five generations of monarch adults were screened for presence of the protozoan parasite, *Ophryocystis elektroscirrha*, in order to eliminate it from the population. *D*. *plexippus* larvae from the maintenance colony were reared on either common milkweed or tropical milkweed, *Asclepias curassavica*. Larvae during the experiments were fed honeyvine milkweed, *Cynanchum laeve* (*syn*. *Ampelamus albidus*) at 25 ± 1°C (16L: 8D), and adults were fed 15% sugar solution.

### Experimental conditions

#### Biotic factors

The different developmental stages including eggs, 1^st^, 2^nd^, 3^rd^, 4^th^ instar larvae (collected at the first day of each instar), pupae, and adults. Tissues, including head, midgut, and carcass, were dissected from the 4^th^ instar larvae. Male and female adults were collected separately to examine expression profiles of candidate genes between sexes.

#### Abiotic factors

To examine temperature influence, 2^nd^ instar *D*. *plexippus* were exposed to 10°C, 22°C, and 37°C for 2 h. For photoperiod, 2^nd^ instars were treated with exposure to 16:8 h, 12:12 h, and 8:16 h light: dark regime for 3 d. For the double-stranded RNA (dsRNA) feeding bioassay, there were three treatments including dsRNAs synthesized from *D*. *plexippus vATPase subunit A* (dsDP), *β-glucuronidase* (GUS) (dsGUS), and water control. Neonates (< 12 h) were given 1.6 μl of water solutions containing 5.0 μg/μl dsRNA each day for two days. Larvae were collected on the third day of the experiment. Altogether, 16μg of dsRNA was ingested by each *D*. *plexippus* larva.

For egg samples, six individuals were collected for each replicate; for pupa samples, one pupa was collected for each replicate. For the other biotic and abiotic conditions, approximately five individuals were collected for each treatment with three replicates. All samples were snap frozen with liquid nitrogen in 1.5 ml centrifuge tubes and then stored at -80°C.

### Total RNA extraction and cDNA synthesis

Total RNA was extracted using TRIzol reagent (Invitrogen, Carlsbad, CA) according to previously described methods [29, 30]. First-strand cDNA was synthesized from 700 ng of total RNA using the M-MLV reverse transcription kit (Invitrogen, Carlsbad, CA) according to manufacturer’s recommendations. The cDNA was diluted 10-fold for the subsequent RT-qPCR studies.

### Reference gene primer design and RT-qPCR

Nine reference genes were selected (Table 1). The primers for the RT-qPCR were designed online (https://www.idtdna.com/Primerquest/Home/Index). The information of RT-qPCR amplification reaction and program were described in detail in our previous study [29, 30]. The standard curve and PCR efficiency of each candidate reference gene were constructed and calculated according to previously described methods [29, 30].

**Table 1:** Primers used for RT-qPCR.

Gene	Accessionnumber	Primer sequences (5’-3’)	Length(bp)	Efficiency(%)	R^2^
*EF1A*	DQ157894	F: TGTCGCTTTCGTACCCATTT	114	97.1	0.989
		R: CCTTCAGCCTTACCCTCTTTAC			
*NADH*	U32457	F: GGTGTTTTAATTGGGGTTGC	122	102.3	0.9992
		R:GCATCAGAAAAAGTTTGTAATAATCC			
*GAPDH*	EU141486	F: CGTTCCCGTAGCTAATGTATCC	93	106.1	0.9985
		R: GCTTCCTTGACCTTCTGTTTAATG			
*CypA*	EHJ70135	F: CAACGGCTCTCAATTCTTCATC	89	99.9	0.9898
		R: CCATGCCTTCAACCACATTAC			
*RPS5*	EU141382	F: GTCGTGTGAACCAGGCTATT	98	100.5	0.9991
		R: GAGCTCATCAGCAACACATTC			
*RP49*	AY971345	F: CCGGAAGGTGTTAGTCCACAAC	71	98.6	0.9997
		R: CGGCGCAGTACTTCCTATTCTG			
*18S*	AF394668	F: TGAGAAACGGCTACCACATC	102	109.3	0.9997
		R: CGTAAGAGTCCCGTATCGTTATT			
*28S*	GQ229491	F: AACAAGTACCGTGAGGGAAAG	94	102.7	0.9978
		R: CATTCGAGTTTCGCAGGTTTAC			
*v-ATPase*	EHJ63113	F: AGGACGACTTCCTGCAACAGAACA	84	105.9	0.9998
		R: TGTTCTTCAACATGCCCACCGTCT			

### Data analysis

Stability of the nine candidate reference genes were evaluated by four computational programs, including *geNorm* [6], *NormFinder* [10], *BestKeeper* [31], and the *ΔC*_*t*_ method [32]. In addition, *RefFinder* (http://www.leonxie.com/referencegene.php), a web-based comprehensive platform, which integrates the four above mentioned algorithms, provided the overall ranking of the stability of these reference genes.

### Validation of selected reference genes

The photoperiod-relevant *tim* gene of *D*. *plexippus* was used to evaluate the candidate reference genes. *tim* expression levels were determined in 2^nd^ instars treated for 3 d under three photoperiod conditions (16L:8D, 12L:12D, and 8L:12D). Relative quantification of *tim* in different samples was performed using the 2^*-ΔΔCt*^ method [33].

## Results

### Performance and *Ct* values of candidate reference genes

Each of the tested genes displayed a single amplicon with the expected size on a 1.5% agarose gel (S1 Fig). Moreover, gene-specific of these candidates was affirmed by a single peak in melting curve analysis (S2 Fig). Standard curves were created for all the candidates, and the PCR efficiency and correlation coefficient for each standard curve were shown in Table 1.

**Fig 1:**
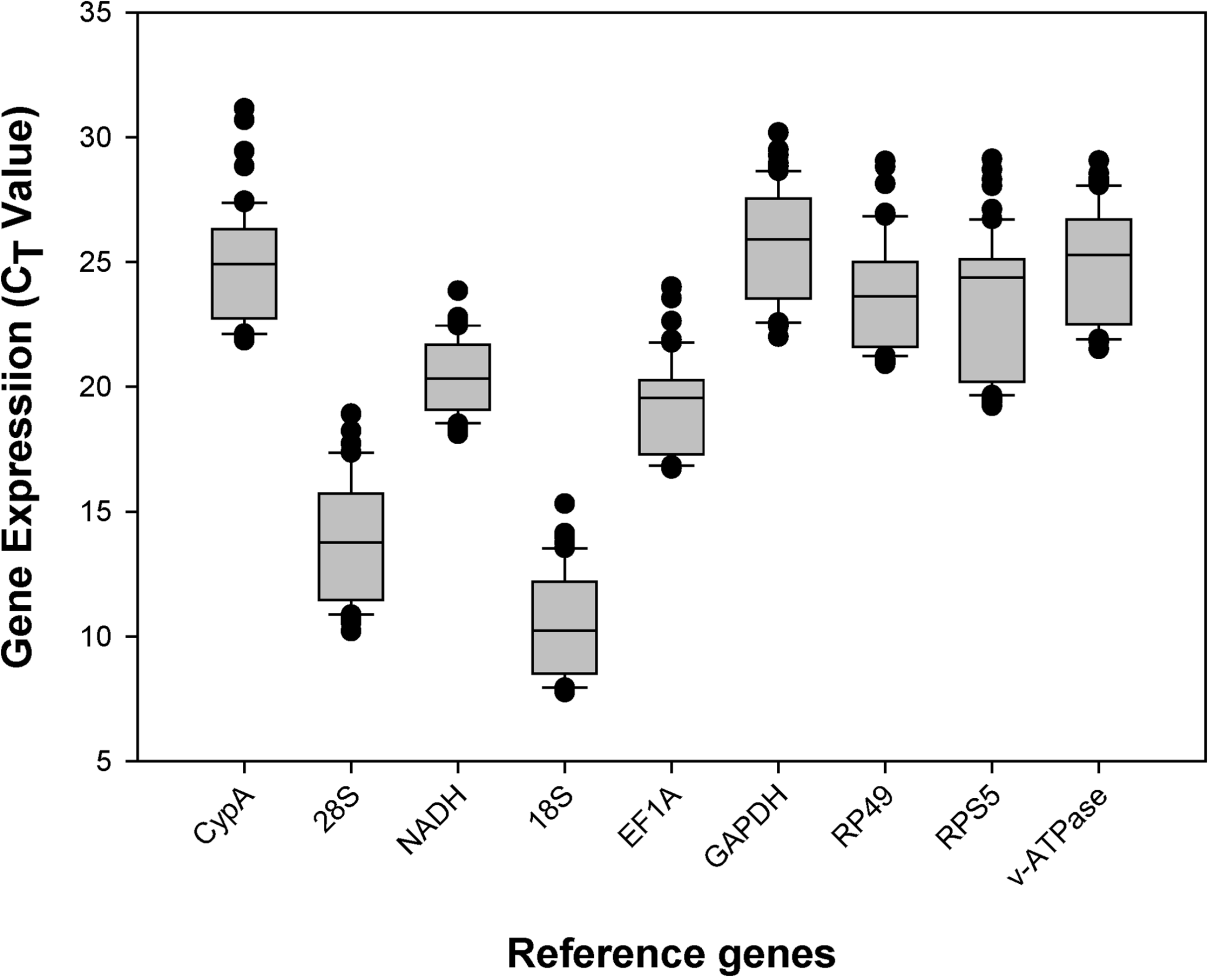
Expression profiles of candidate reference genes in *D*. *plexippus*. The expression level of candidate reference genes in 60 tested samples are documented in *C*_*t*_-value. The dot indicates the maximum or minimum value of replicated samples, while whiskers indicate the standard error of the mean.

Gene expression analyses of the nine reference genes exhibited a broad *C*_*t*_ range (Fig 1). *C*_*t*_ values ranged from 7 to 29, while most of the values were distributed between 11 and 22. The most abundant transcripts were *18S* and *28S*; and the least abundant transcripts were *GAPDH* and *CypA*.

### Stability of candidate reference genes under biotic conditions

#### Developmental stage

All software programs, except *BestKeeper*, identified *EF1A* as the most stable gene (Table 2).

**Table 2:** Stability of reference gene expression under biotic conditions.

Biotic	Gene	*geNorm*		*Normfinder*		*BestKeeper*		*ΔCt*	
conditions		Stability	Rank	Stability	Rank	Stability	Rank	Stability	Rank
Development	*EF1A*	0.53	1	0.61	1	1.35	6	1.07	1
	*NADH*	1.27	8	1.66	9	0.89	2	1.81	9
	*GAPDH*	1.12	7	0.93	7	0.98	4	1.33	8
	*RP49*	0.53	1	0.83	5	1.20	9	1.14	4
	*18S*	0.91	5	0.81	4	0.91	3	1.24	5
	*28S*	0.70	3	0.65	2	1.39	7	1.10	2
	*v-ATPase*	1.04	6	0.90	6	0.85	1	1.30	7
	*CypA*	0.80	4	0.99	8	1.40	8	1.30	6
	*RPS5*	0.66	2	0.75	3	1.20	5	1.14	3
Tissue	*EF1A*	0.45	1	0.51	2	0.80	7	0.87	2
	*NADH*	0.60	3	0.71	5	0.77	5	0.98	3
	*GAPDH*	1.01	8	1.22	9	0.73	4	1.37	9
	*RP49*	0.57	2	0.75	7	0.73	3	1.00	5
	*18S*	0.80	5	0.82	8	1.03	9	1.07	8
	*28S*	0.45	1	0.20	1	0.53	2	0.79	1
	*v-ATPase*	0.70	4	0.67	3	0.97	8	0.99	4
	*CypA*	0.91	7	0.68	4	0.41	1	1.00	6
	*RPS5*	0.87	6	0.75	6	0.77	6	1.05	7
Sex	*EF1A*	0.41	3	0.64	5	0.90	6	0.98	4
	*NADH*	1.23	8	2.21	9	1.45	9	2.27	9
	*GAPDH*	0.71	6	0.53	4	0.72	3	1.15	6
	*RP49*	0.35	2	0.10	1	0.91	4	0.89	1
	*18S*	0.29	1	0.15	2	0.68	2	0.89	2
	*28S*	0.29	1	0.35	3	0.82	5	0.93	3
	*v-ATPase*	0.93	7	1.24	7	0.64	1	1.55	8
	*CypA*	0.56	5	1.31	8	1.27	8	1.39	7
	*RPS5*	0.43	4	0.70	6	0.91	7	1.01	5

**Fig 2:**
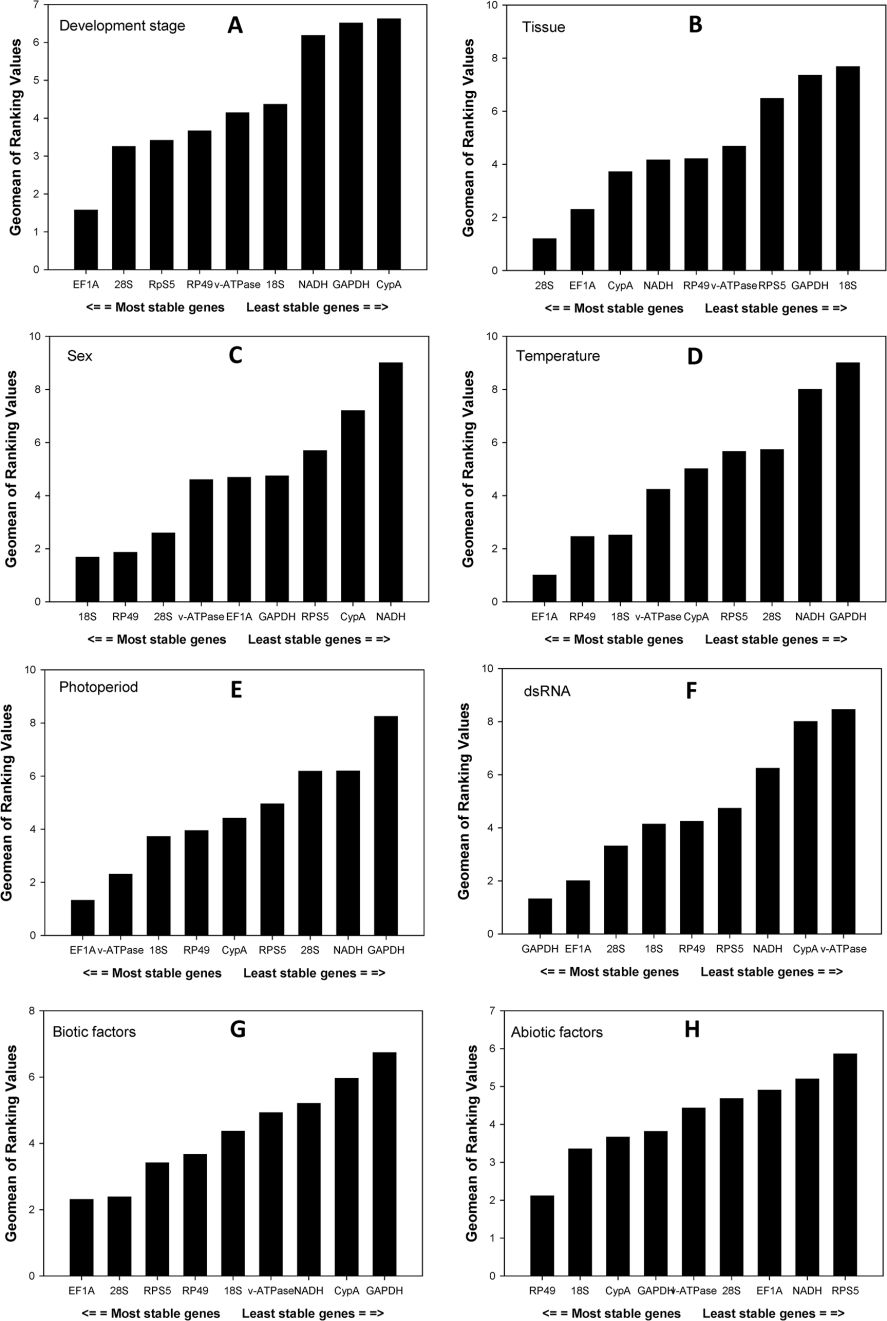
Stability of candidate reference genes expression under different treatment by *RefFinder*. A lower *Geomean* value indicates more stable expression.

**Fig 3:**
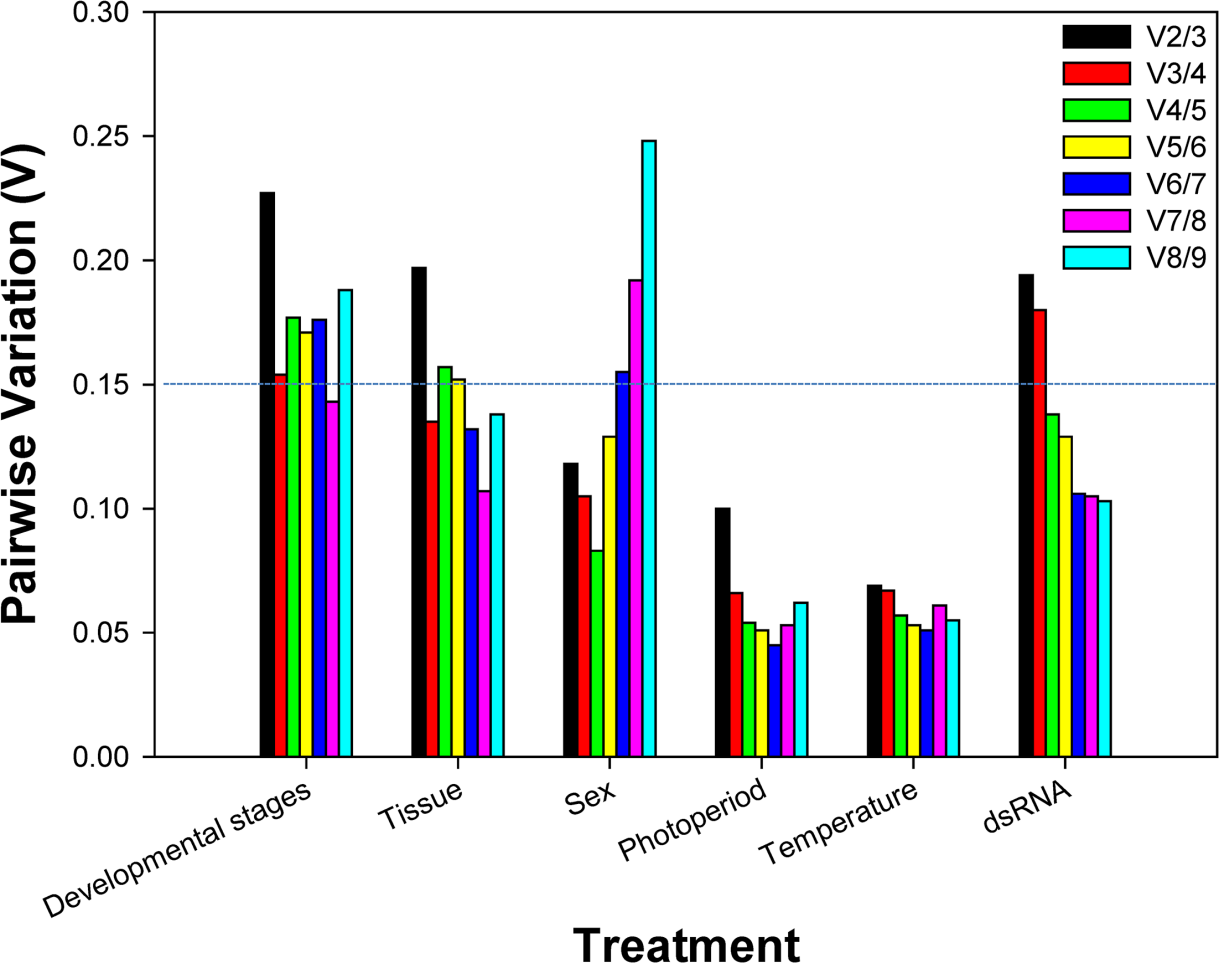
Determination of the optimal number of reference genes. Pairwise variation (V) is an index for determining the optimal number of reference genes for accurate RT-qPCR normalization. A cut-off value for pairwise variation of 0.15 was recommended [6].

According to *RefFinder*, the overall order from the most stable to the least stable reference genes across different developmental stages was: *EF1A*, *28S*, *RPS5*, *RP49*, *v-ATPase*, *18S*, *NADH*, *GAPDH*, *CypA* (Fig 2A). *geNorm* analysis revealed that the pair-wise variation value V3/4 was 0.154, close to the proposed 0.15 cut-off (Fig 3), suggesting that three reference genes were required for reliable normalization throughout developmental stages.

#### Tissue

Majority of the software programs ranked *28S* as the most stable gene (Table 2). Based on *RefFinder*, the overall order from the most to the least stable reference genes in different tissue was: *28S*, *EF1A*, *CypA*, *NADH*, *RP49*, *v-ATPase*, *RPS5*, *GAPDH*, *18S* (Fig 2B). *geNorm* analysis revealed that the first V-value < 0.15 showed at V3/4 (Fig 3), suggesting that three reference genes were sufficient for reliable normalization in different tissue types.

#### Sex

According to the results generated by *geNorm*, *18S* was ranked as the most stable gene whereas the other three programs identified it as the second stable gene (Table 2). According to *RefFinder*, the overall order from the most stable to the least stable reference genes in both female and male adults was: *18S*, *RP49*, *28S*, *v-ATPase*, *EF1A*, *GAPDH*, *RPS5*, *CypA*, *NADH* (Fig 2C). *geNorm* analysis revealed that the first V-value < 0.15 showed at V2/3 (Fig 3), suggesting that two reference genes were enough for accurate normalization in female and male adults.

### Stability of candidate reference genes under abiotic conditions

#### Temperature

All software programs identified *EF1A* as the most stable gene (Table 3). According to *RefFinder*, the overall order from the most stable to the least stable reference genes under the temperature stress was: *EF1A*, *RP49*, *18S*, *v-ATPase*, *CypA*, *RPS5*, *28S*, *NADH*, *GAPDH* (Fig 2D). *geNorm* analysis revealed that first V-value < 0.15 showed at V2/3 (Fig 3), suggesting that two reference genes were sufficient for accurate normalization across different temperatures.

**Table 3:** Stability of reference gene expression under abiotic conditions.

Abiotic	Gene	*geNorm*		*Normfinder*		*BestKeeper*		*ΔCt*	
conditions		Stability	Rank	Stability	Rank	Stability	Rank	Stability	Rank
Temperature	*EF1A*	0.18	1	0.05	1	0.15	1	0.31	1
	*NADH*	0.38	7	0.45	8	0.39	8	0.53	8
	*GAPDH*	0.42	8	0.49	9	0.45	9	0.55	9
	*RP49*	0.18	1	0.25	4	0.19	3	0.37	3
	*18S*	0.28	4	0.17	2	0.16	2	0.35	2
	*28S*	0.30	5	0.30	6	0.28	5	0.42	6
	*v-ATPase*	0.25	3	0.26	5	0.20	4	0.39	4
	*CypA*	0.33	6	0.24	3	0.29	6	0.40	5
	*RPS5*	0.21	2	0.37	7	0.29	7	0.44	7
Photoperiod	*EF1A*	0.27	2	0.14	1	0.27	1	0.35	1
	*NADH*	0.32	5	0.29	7	0.40	5	0.40	7
	*GAPDH*	0.37	7	0.43	8	0.46	8	0.51	8
	*RP49*	0.28	3	0.25	4	0.31	3	0.39	5
	*18S*	0.17	1	0.27	6	0.46	8	0.39	4
	*28S*	0.42	8	0.55	9	0.29	2	0.60	9
	*v-ATPase*	0.17	1	0.20	2	0.46	7	0.35	2
	*CypA*	0.33	6	0.23	3	0.41	6	0.39	3
	*RPS5*	0.30	4	0.25	5	0.38	4	0.40	6
dietary RNAi	*EF1A*	0.37	1	0.47	2	0.54	4	0.79	2
	*NADH*	0.81	6	0.65	6	0.62	6	0.91	6
	*GAPDH*	0.37	1	0.46	1	0.51	3	0.79	1
	*RP49*	0.53	2	0.55	4	0.72	9	0.84	3
	*18S*	0.77	5	0.66	7	0.31	1	0.92	7
	*28S*	0.72	4	0.55	3	0.31	2	0.85	4
	*v-ATPase*	0.91	8	0.90	9	0.69	7	1.08	9
	*CypA*	0.86	7	0.87	8	0.69	8	1.05	8
	*RPS5*	0.66	3	0.63	5	0.58	5	0.90	5

#### Photoperiod

All software programs identified *EF1A* as the most or second to most stable gene (Table 3). According to *RefFinder*, the overall order from the most stable to the least stable reference genes under the photoperiod stress was: *EF1A*, *v-ATPase*, *18S*, *RP49*, *CypA*, *RPS5*, *28S*, *NADH*, *GAPDH* (Fig 2E). *geNorm* analysis revealed that first V-value < 0.15 showed at V2/3 (Fig 3), suggesting that two reference genes were adequate for accurate normalization under the photoperiod condition.

#### dietary RNAi

All software programs, except *BestKeeper*, identified *GAPDH* as the most stable gene (Table 3). According to *RefFinder*, the overall order from the most stable to the least stable reference genes under the dietary RNAi treatment was: *GAPDH*, *EF1A*, *28S*, *18S*, *RP49*, *RPS5*, *NADH*, *CypA*, *v-ATPase* (Fig 2F). *geNorm* analysis revealed that the pair-wise variation value V4/5 was below the proposed 0.15 cut-off (Fig 3). This result suggests that four reference genes were required for accurate normalization under the dietary RNAi condition.

According to *RefFinder*, the overall order from the most stable to the least stable reference genes under biotic conditions was: *EF1A*, *28S*, *RPS5*, *RP49*, *18S*, *v-ATPase*, *NADH*, *CypA*, *GAPDH* (Fig 2G); and the overall order from the most stable to the least stable reference genes under abiotic conditions was: *RP49*, *18S*, *CypA*, *GAPDH*, *v-ATPase*, *28S*, *EF1A*, *NADH*, *RPS5* (Fig 2H).

### Validation of selected reference genes

**Fig 4:**
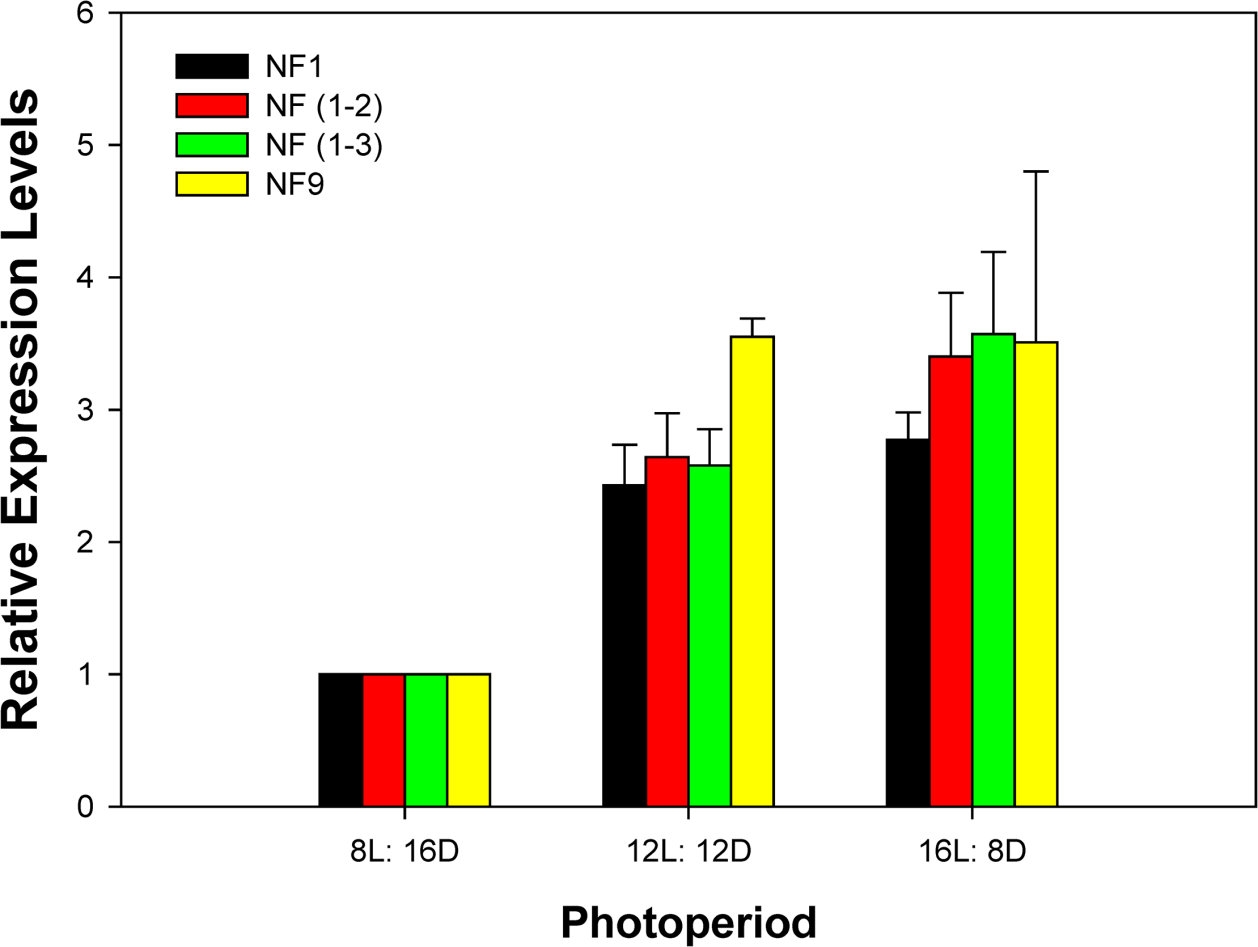
Validation of recommended reference genes. Expression profiles of *tim* gene under three photoperiod conditions were investigated using different combinations of reference genes. NF1, NF (1–2), NF (1–3), and NF9 indicate that the expression of *tim* was normalized using the best, the top two, the top three, or, the worst reference genes, respectively. Bar represents the mean and standard error of three biological replicates.

Using two best reference gene combinations for normalization, two genes [*EF1A* and *v-ATPase*; NF (1–2)] or three genes [*EF1A*, *v-ATPase*, and *18S*; NF (1–3)], similar expression levels of *tim* occurred within each photoperiod (Fig 4). Under the 12L: 12D photoperiod condition, the expression level of *tim* was the highest when the reference gene with the highest *Geomean* value (*GAPDH*; NF9) was used (Fig 4).

## Discussion

The monarch butterfly is commonly used as a non-target insect for the ecological risk assessment of transgenic crops in the U.S.; plus interest in the migratory biology and genetics of this insect suggest future post-genome studies will occur. In this study, expression profiles of nine candidate reference genes from *D*. *plexippus* were evaluated under diverse experimental conditions. This research is the first step toward establishing a standardized RT-qPCR analysis for this insect.

Our results demonstrate that the best-suited reference genes can vary depending on biotic and abiotic factors (Tables 2 and 3). Thus, reference genes need to be selected based on the experimental conditions. Unfortunately, a universal reference gene that covers all experimental conditions is unrealistic. For example, *EF1A*, which plays an important role in translation by catalyzing the GTP-dependent binding of aminoacyl-tRNA to the acceptor site of the ribosome, is the most stable reference gene for biotic factors (Fig 2G). This result is consistent with previous studies in other insects [34, 35]. However, *EF1A* ranked poorly (7^th^) as a reference gene for abiotic factors (Fig 2H).

To avoid biased normalization, many researchers have started to advocate the use of multiple reference genes to analyze gene expression [4, 5, 9, 11, 12, 29, 30, 36]. Our results demonstrate that two reference genes are required for reliable normalization under photoperiod, temperature, and sex conditions; three reference genes are required for reliable normalization in different tissue types and under different developmental stages; four reference genes are sufficient to normalize the expression and provide a more conservative estimation of target gene expression under dietary RNAi condition (Fig 3). As a result, we suggest that using different combinations of reference genes are necessary for studying gene expression in *D*. *plexippus*, especially with variable experimental conditions.

This work represents an important first step toward establishing a standardized gene analysis framework for *D*. *plexippus*. Analysis of our data indicate that *EF1A* and *RP49* are the most stable reference genes, respectively, under biotic (development, tissue, and sex) and abiotic (photoperiod, temperature, and dietary RNAi) conditions. With the recent release of *D*. *plexippus* genome, results from this study provide a critical piece for the subsequent genomics and functional genomics research in this emerging insect model, and shed light on the ecological risk assessment of RNAi-based biopesticides on this non-target bio-indicator agent.

## Supporting Information

S1 Fig The agrose gel profile of the nine candidate reference genes. M, EZ Load 100 bp Molecular Ruler; Templates in the PCR reactions were as follows: 1) *EF1A*, 2) *NADH*, 3) *GAPDH*, 4) *CypA*, 5) *RPS5*, 6) *RP49*, 7) *18S*, 8) *28S*, and 9) *v-ATPase*.
(TIFF)

S2 Fig Melting curve of the nine candidate reference genes. (TIFF)
