# Supplementary figures and images for: Selection of Reference Genes for RT-qPCR Analysis in the Monarch Butterfly, Danaus plexippus (L.), a Migrating Bio-Indicator
Source: PLoS One. 2015 Jun 1;10(6):e0129482. doi: 10.1371/journal.pone.0129482 (PMC4452232; doi:10.1371/journal.pone.0129482)

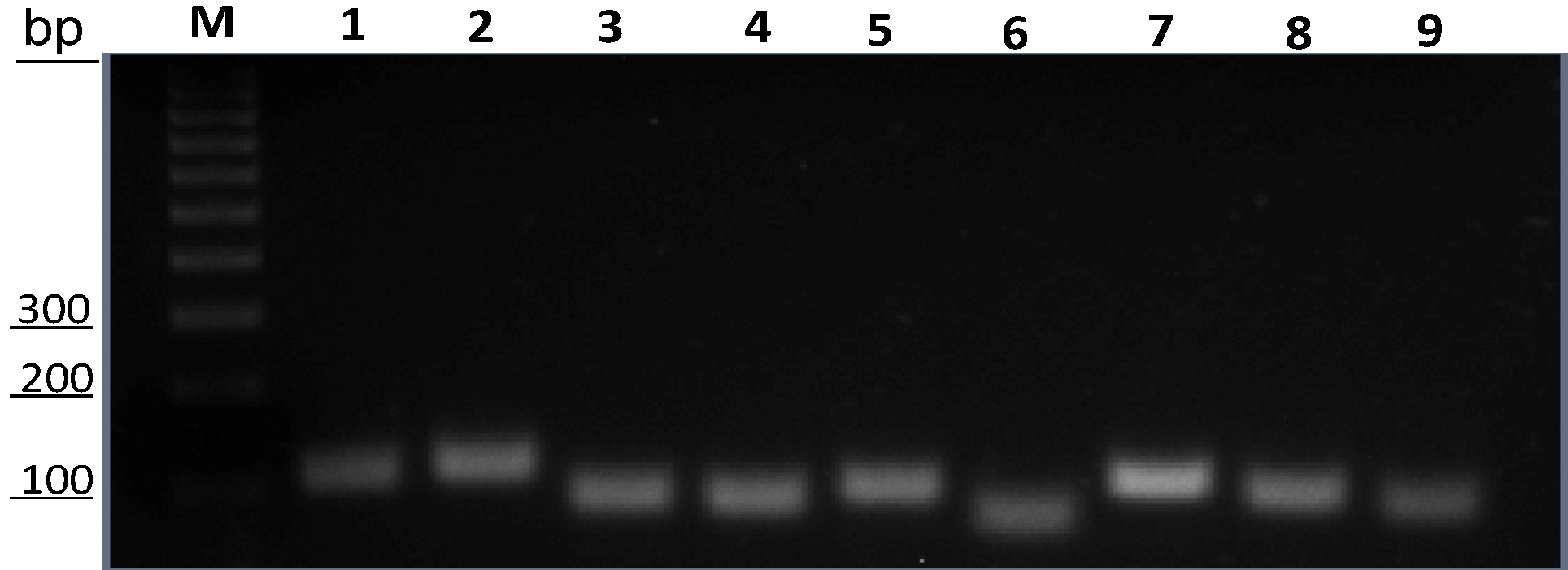

Supplement: S1 Fig — M, EZ Load 100 bp Molecular Ruler; Templates in the PCR reactions were as follows: 1) EF1A, 2) NADH, 3) GAPDH, 4) CypA, 5) RPS5, 6) RP49, 7) 18S, 8) 28S, and 9) v-ATPase. (TIFF) [file pone.0129482.s001.tiff]

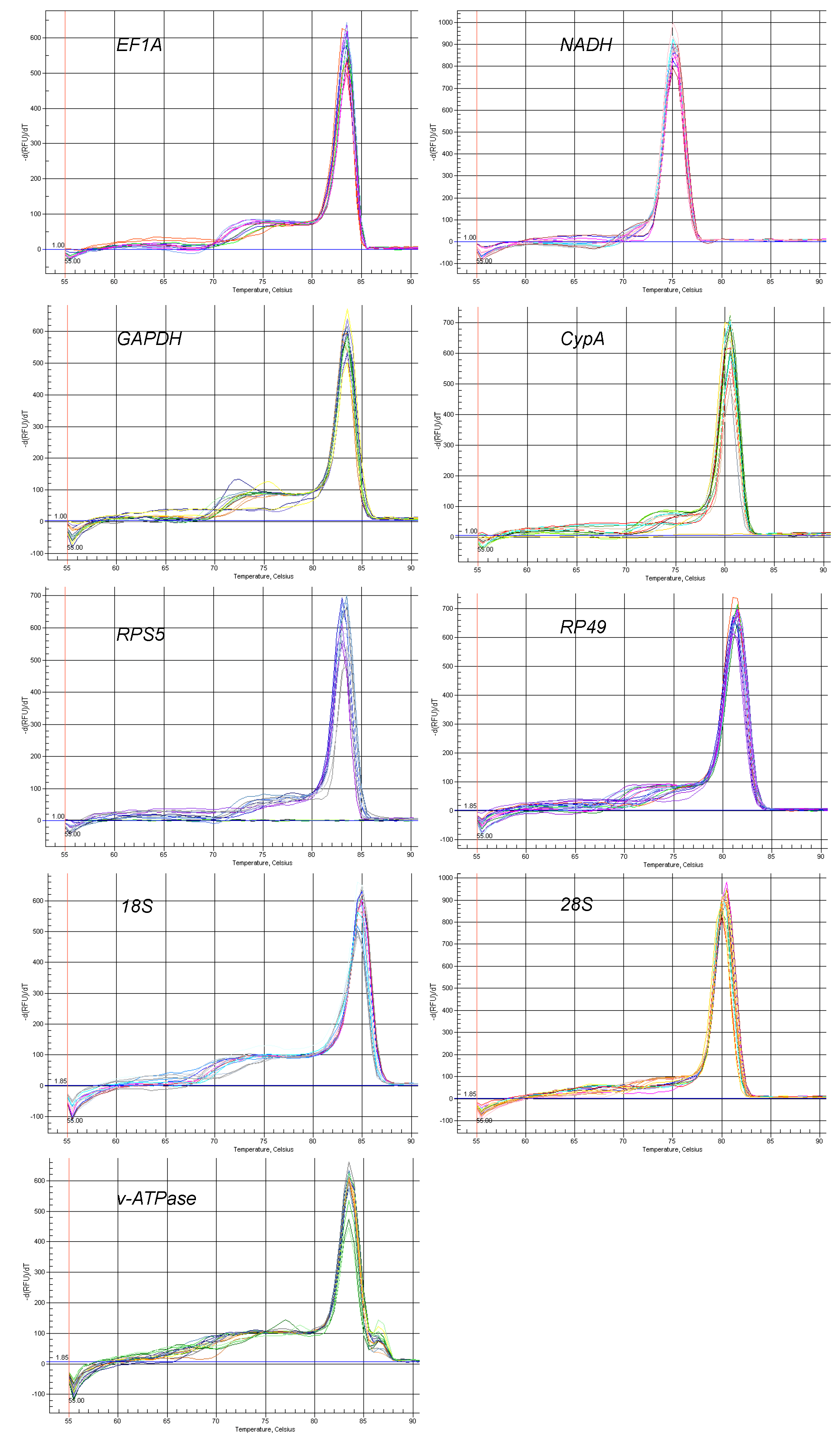

Supplement: S2 Fig — (TIFF) [file pone.0129482.s002.tiff]
